# Comparative Analysis of Hiatal Hernia Repair Techniques: A Meta-Analysis Review Study on Biological Mesh, Phasix™ Mesh, and Primary Repair

**DOI:** 10.7759/cureus.82201

**Published:** 2025-04-13

**Authors:** Samer Ganam, Chandler N Lentovich, Ryan Tang, Rahul Mhaskar, Joseph A Sujka, Christopher G DuCoin, Emily Coughlin

**Affiliations:** 1 Surgery, Tampa General Hospital, Tampa, USA; 2 Surgery, University of South Florida Morsani College of Medicine, Tampa, USA; 3 Medicine, University of South Florida Morsani College of Medicine, Tampa, USA; 4 Internal Medicine and Medical Education, University of South Florida Morsani College of Medicine, Tampa, USA; 5 Biostatistics, University of South Florida Morsani College of Medicine, Tampa, USA

**Keywords:** biological mesh, hiatal hernia, phasix™ st mesh, primary repair, recurrence rate, suture cruroplasty

## Abstract

Mesh usage in hiatal hernia repair is debated regarding recurrence rates and complications. This study aims to compare the efficacy of Phasix™ ST mesh, biological mesh, and primary repair in terms of recurrence rates, reoperation rates, and mesh-related complications. Preferred Reporting Items for Systematic Reviews and Meta-Analyses (PRISMA) guidelines were followed to search literature in PubMed, Embase, and Web of Science from January 2011 to November 2023. Included studies focused on participants aged 18+ undergoing hiatal hernia repair with specific mesh types or repair methods. Data on recurrence rates, reoperation rates, and mesh-related complications were analyzed by BMI and follow-up time subgroups. Statistical analysis used the Mantel-Haenszel random-effects model. Bias in studies was assessed using the ROBINS-I and Cochrane risk of bias tools for non-randomized and randomized trials. Twenty-two studies involving 2,008 patients were included. A double-arm meta-analysis comparing biological mesh and suture cruroplasty found no significant difference in recurrence or reoperation rates. The randomized trial showed no significant difference in recurrence (OR 2.02; 95% CI 0.71-5.76) or reoperation (OR 0.71; 95% CI 0.17-2.96). Non-randomized studies also showed no significant difference in recurrence (OR 0.32; 95% CI 0.03-3.06) or reoperation (OR 0.35; 95% CI 0.05-2.37). In single-arm meta-analyses, Phasix™ ST mesh had the lowest recurrence rate, followed by biological mesh and suture cruroplasty. No reoperations were reported with Phasix™ ST mesh. Postoperative dysphagia was lowest with Phasix™ ST mesh. In conclusion, Phasix™ ST mesh showed the lowest recurrence, reoperation rates, and dysphagia compared to biological mesh and primary repair, making it a preferred option.

## Introduction and background

A hiatal hernia is characterized by the displacement of the upper stomach and possibly an additional organ, such as the colon, small intestine, or spleen, into the thoracic cavity via a widening of an esophageal hiatus within the diaphragm [1].

Hiatal hernias may be congenital or acquired, and loss of muscle strength and elasticity with increased age may be a risk factor. Other risk factors include conditions that increase intra-abdominal pressure, such as obesity, pregnancy, chronic constipation, and chronic obstructive pulmonary disease (COPD). Hiatal hernias typically present with similar symptoms to gastroesophageal reflux disease (GERD), such as heartburn, regurgitation, and chronic cough. To diagnose whether a patient has a hiatal hernia, endoscopy, manometry, pH monitoring, and/or esophagography should be performed.

Recommendations regarding when to perform a hiatal hernia repair depend on both the presence of symptoms and hernia size. Any patient experiencing symptoms secondary to the hiatal hernia, such as heartburn, regurgitation, or difficulty swallowing, is recommended to undergo hiatal hernia repair. In addition, patients who are asymptomatic with a large hiatal hernia, such as one in which over half of the stomach protrudes through the hiatus, are also recommended to undergo hiatal hernia repair if they are under the age of 60 with no severe comorbidities. Even after repair, hiatal hernias tend to have a high recurrence rate. Surgeons have tried to reduce the recurrence rate of hiatal hernias by reinforcing suture repair with prosthetic mesh. Still, erosion and shrinkage of the mesh have led the surgical community to explore other ways to strengthen the surgical repair [2,3]. Bioabsorbable mesh has been suggested as an adjunct to surgical repair, and studies have suggested that it is safe and has a low complication rate [4].

One bioabsorbable mesh of interest is Phasix™ ST mesh. Other institutions have reported that Phasix™ ST mesh is not associated with mesh-related complications and is more effective in reducing hiatal hernia recurrence in both short- and medium-term follow-ups when compared to simple suture cruroplasty [5-9]. However, there is limited literature that directly compares the use of Phasix™ ST mesh to biological mesh, and typically, if there is an attempt at comparing the two, it is inconclusive due to limited data [9]. Therefore, in this paper, we will compare patient outcomes, including recurrence rate and mesh type-related complications, between those who have undergone hiatal hernia repair using biological mesh and those who have undergone hiatal hernia repair using Phasix™ ST bioabsorbable mesh and both with primary repair (suture cruroplasty).

Therefore, the objective of this study is to determine whether there is a significant difference in hiatal hernia recurrence rate and reoperation between suture cruroplasty, biological mesh, or Phasix™ ST mesh usage in hiatal hernia repair. 

## Review

Methods

The nature of our meta-analysis study exempted us from requiring Institutional Review Board (IRB) approval. A literature search was performed by the Preferred Reporting Items for Systematic Reviews and Meta-Analyses (PRISMA) guidelines. PubMed, Embase, and Web of Science were queried for records between January 2011 and November 2023 using relevant search terms. Exclusion criteria included case reports, case series, conference abstracts, non-human studies, specific populations (neonatal patients), non-English records, and studies where patients had secondary or concomitant surgery. Inclusion criteria were studies with participants 18 or older who underwent primary hiatal hernia repair using Phasix™ ST mesh, biological mesh, or primary repair.

Data Extraction

The following data were extracted from each study: study design type, follow-up time, number of patients, mean age, mesh type, hernia size, recurrence events/rates, reoperation events/rates, mean BMI, complications, GERD, and dysphagia. BMI was divided into two groups: the "Grade 1" group consisted of patients with a BMI of 25-29, and the "Grade 2" group consisted of patients with a BMI of 30+. Groups were assessed in terms of the following follow-up times: 3-12 months, 14-18 months, 24-40 months, and greater than four years. Hernia size was not included in the statistical analysis due to variability in reporting. To assess the risk of bias, we used the Cochrane risk of bias tool for randomized controlled trials and the ROBINS-I risk of bias tool for non-randomized trials.

Statistical Methods

Meta-analysis of studies comparing biological mesh and cruroplasty was conducted using RevMan Version 5.4 (The Cochrane Collaboration, London, England, United Kingdom). The Mantel-Haenszel random-effects model reported odds ratios (OR) and 95% confidence intervals (95% CI). Single-arm studies were pooled using a random-effects model reporting pooled proportions and 95% CI. Single-arm analysis was conducted using Stata Statistical Software: Release 17.0 (April 2021; StataCorp LLC, College Station, Texas, United States). All analyses were assessed for heterogeneity using the I2 statistic.

Results

**Figure 1:**
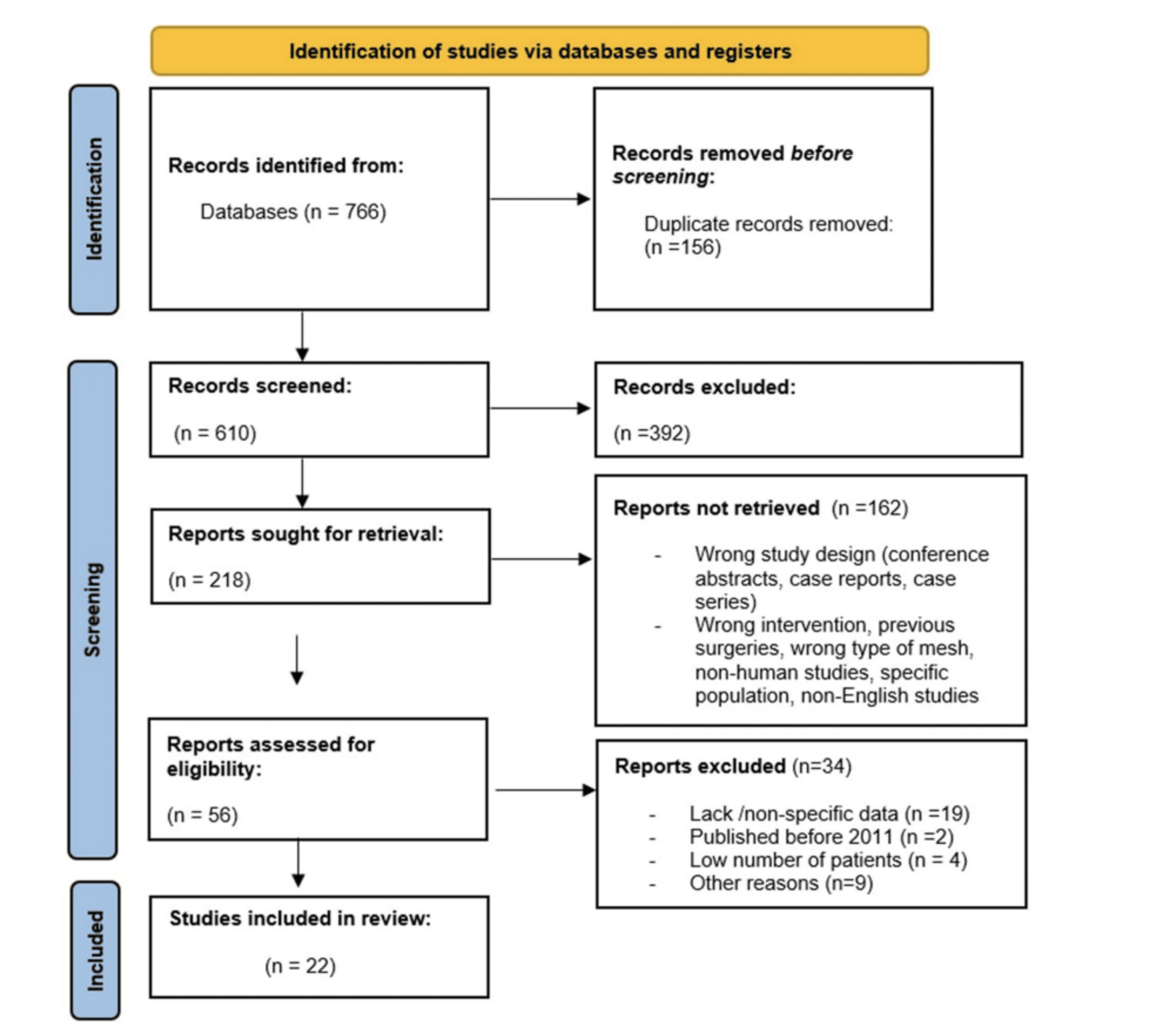
PRISMA diagram PRISMA: Preferred Reporting Items for Systematic Reviews and Meta-Analyses

Our initial search yielded 766 records, and 22 studies were deemed eligible for inclusion (Figure 1). Overall, a total of 2,008 patients who received different types of mesh (211 Phasix™ ST mesh, 1,168 biological mesh, and 629 cruroplasty) were assessed (Tables 1-5).

**Table 1:** Studies used for randomized double-arm meta-analysis

	Authors	Year	Mesh subtype	N	Age	BMI	Median follow-up	Recurrence	Reoperation	Postoperative dysphagia N (%)
Cruroplasty data	Watson et al. [3]	2020	NA	28	Mean age: 67.8 (range: 64.7-70.9)	Mean BMI: 29.6 (range: 28.0-31.2)	3-4 years	39.29%	17.86%	5 (17.86%)
Biological mesh data	Watson et al. [3]	2020	Surgisis mesh	30	Mean age: 68.0 (range: 65.1-70.9)	BMI 29.4 (range: 27.8-31.0)	3-4 years	56.67%	13.33%	3 (10%)

**Table 2:** Studies used for non-randomized double-arm meta-analysis SIS: small intestinal submucosa

Repair type	Authors	Year	Mesh subtype	N	Age	BMI	Median follow-up	Recurrence	Reoperation	Postoperative dysphagia N (%)
Cruroplasty	Oelschlager et al. [10]	2011	NA	39	Mean age: 63 (SD: 10)	Mean BMI: 31.3 (SD: 4.9)	58 months	51.28%	5.13%	0 (0%)
Cruroplasty	Schmidt et al. [11]	2014	NA	32	Mean age: 41 (SD: NA)	Mean BMI: 29.5 (SD: NA)	12 months	15.63%	6.25%	1 (3.13%)
Biological mesh	Oelschlager et al. [10]	2011	SIS mesh	33	Mean age: 64 (SD: 10)	Mean BMI: 30.2 (SD: 5.6)	58 months	42.42%	3.03%	0 (0%)
Biological mesh	Schmidt et al. [11]	2014	Human acellular dermal matrix	38	Mean age: 51 (SD: NA)	Mean BMI 31.37 (SD: NA)	12 months	0%	0%	2 (5.26%)

**Table 3:** Studies used for Phasix™ ST mesh single-arm meta-analysis

Authors	Year	N	Age	BMI	Median follow-up	Recurrence	Reoperation	Postoperative dysphagia N (%)
Abdelmoaty et al. [12]	2020	50	Median age: 67 (range: 44-84)	Mean BMI: 30.6 (range: 20-41.5)	12 months	8%	0%	5 (10%)
Aiolfi et al. [5]	2022	68	Mean age: 66.3 (SD: 12.7)	Mean BMI: 26.3 (SD: 5.1)	27 months	8.82%	0%	2 (2.94%)
Konstantinidis and Charisis [7]	2023	30	Median age: 56 (range: 27-81)	Median BMI: 27.5 (range: 21-38.5)	14 months	0%	0%	0 (0%)
Panici Tonucci et al. [6]	2020	63	Mean age: 68.2 (SD: 23.2)	Mean BMI: 26.9 (SD: 3.5)	17 months	3.17%	0%	NA

**Table 4:** Studies used for biological mesh single-arm meta-analysis

Authors	Year	Mesh subtype	N	Age	BMI	Median follow-up	Recurrence	Reoperation	Postoperative dysphagia N (%)
Armijo et al. [2]	2021	AlloDerm human tissue matrix	152	Median age: 60 (range: 49-69)	Median BMI: 29.44 (range: 26.82-32.30)	27 months	44.08%	NA	52 (85.25%)
Armijo et al. [2]	2021	Strattice porcine tissue matrix	42	Median age: 62 (range: 58-74)	Median BMI: 29.74 (range: 25.86-34.43)	27 months	40.48%	NA	12 (80%)
Bell et al. [13]	2013	AlloGraft dermal matrix	252	Mean age: 57 (SD: 13.4)	Mean BMI: 30.0 (SD: 5.7)	18 months	9.5%	NA	NA
Chang and Thackeray [14]	2016	Veritas Collagen Matrix	221	Mean age: 51.1 (SD: 12.7)	Mean BMI: 36.1 (SD: 7.1)	14.5 months	3.62%	0.45%	56 (25.34%)
Korwar et al. [15]	2019	Surgisis mesh	154	Mean age: 65 (SD: 12)	NA	35 months	16.23%	3.25%	NA
Lidor et al. [16]	2015	Veritas Collagen Matrix	70	Mean age: 61.5 (SD: 13.5)	Mean BMI: 30.06 (SD: 6.74)	12 months	27.14%	5.71%	NA
Lomelin et al. [17]	2017	Strattice biologic mesh	35	Mean age: 63.1 (SD: 12.0)	Mean BMI: 30.8 (SD: 6.3)	12 months	14.29%	0%	1 (2.86%)
Rosen et al. [18]	2019	MIROMESH derived from decellularized porcine liver	27	Mean age: 63.3 (range: 26-79)	BMI: 30.7 (range: 22-39)	24 months	11.11%	0%	1 (3.7%)
Shrestha et al. [19]	2019	Veritas Collagen Matrix	60	Median age: 71 (range: 42-89)	Median BMI: 29 (range: 19-42)	60 months	23.33%	1.67%	5 (8.33%)
Ward et al. [20]	2015	Acellular human dermis	54	Mean age: 60.8±10.5 for the FlexHD group and 64.3±10.1 for the AlloDerm group	Mean BMI: 31.3±4.66 for the FlexHD group and 32.6±6.55 for the AlloDerm group	33 months	14.8%	7.41%	NA

**Table 5:** Studies used for suture cruroplasty single-arm meta-analysis

Authors	Year	N	Age	BMI	Median follow-up	Recurrence	Reoperation	Postoperative dysphagia N (%)
Asti et al. [21]	2016	43	Mean age: 65.8 (SD: 13.6)	Mean BMI: 26.5 (SD: 3.3)	24 months	18.6%	0%	1 (2.33%)
Dallemagne et al. [22]	2011	21	Median age of 66 (range: 37-85 years)	NA	151 months	66.67%	0%	3 (14.29%)
Chan et al. [23]	2022	49	Mean age: 57.3 (SD: 10.7)	NA	77.1 months	20.41%	16.33%	2 (4.08%)
Gouvas et al. [24]	2011	48	Median age: 65 (range: 38-84 years)	NA	12 months	8.33%	0%	NA
Koetje et al. [25]	2017	127	Mean age: 66.9 (SD: 10.3)	Mean BMI: 28.7 (SD: 4.2)	39.3 months	23.62%	6.3%	NA
Mohr et al. [26]	2023	242	Mean age: 51.1 (SD: 13.0)	Mean BMI: 29.7 (SD: 5.6)	17 months	16.53%	6.2%	32 (13.22%)

**Figure 2:**
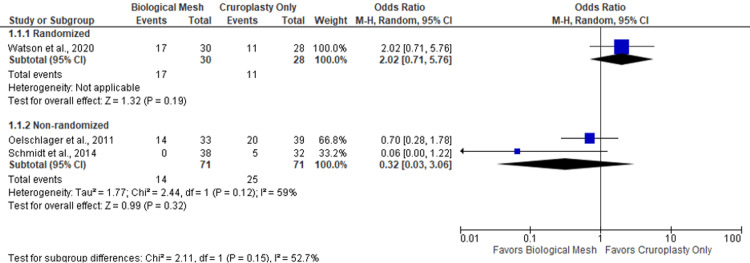
Double-arm meta-analysis, recurrence rate

A double-arm meta-analysis was done with three studies [3,10,11] comparing biological mesh (N=101) and cruroplasty (N=99). One trial was randomized and showed no evidence of a significant difference in recurrence in patients with biological mesh vs. cruroplasty (OR 2.02; 95% CI 0.71-5.76). Two studies were non-randomized, and they showed no evidence of a significant difference in recurrence among biological mesh vs. cruroplasty (OR 0.32; 95% CI 0.03-3.06). This analysis did not show significant heterogeneity (I2=59%; p=0.12) (Figure 2) (Tables 1-2).

**Figure 3:**
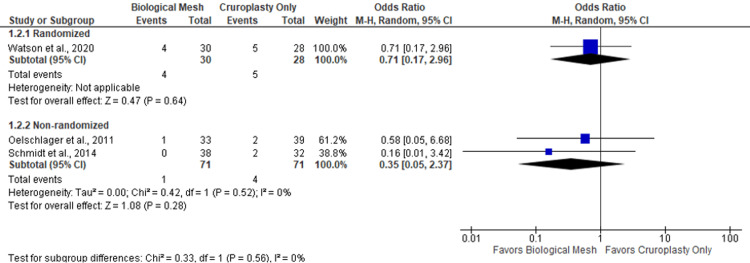
Double-arm meta-analysis, reoperation rate

Additionally, the randomized and non-randomized trials comparing biological mesh vs. cruroplasty showed no evidence of a significant difference in reoperations (OR 0.71; 95% CI 0.17-2.96 and OR 0.35; 95% CI 0.05-2.37, respectively). This analysis did not show significant heterogeneity (I2=0%; p=0.52) (Figure 3).

**Figure 4:**
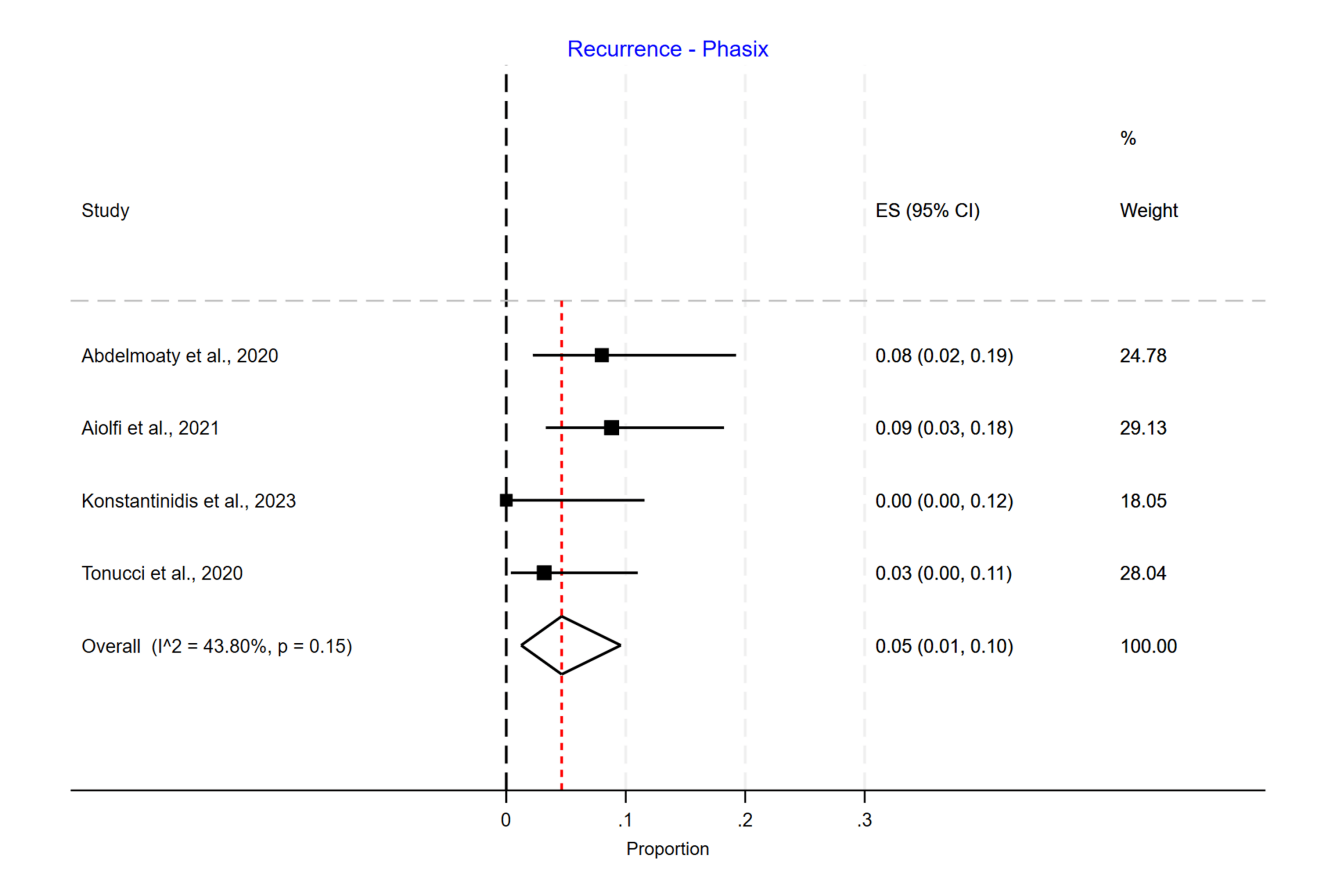
Recurrence rate of Phasix™ mesh studies

**Figure 5:**
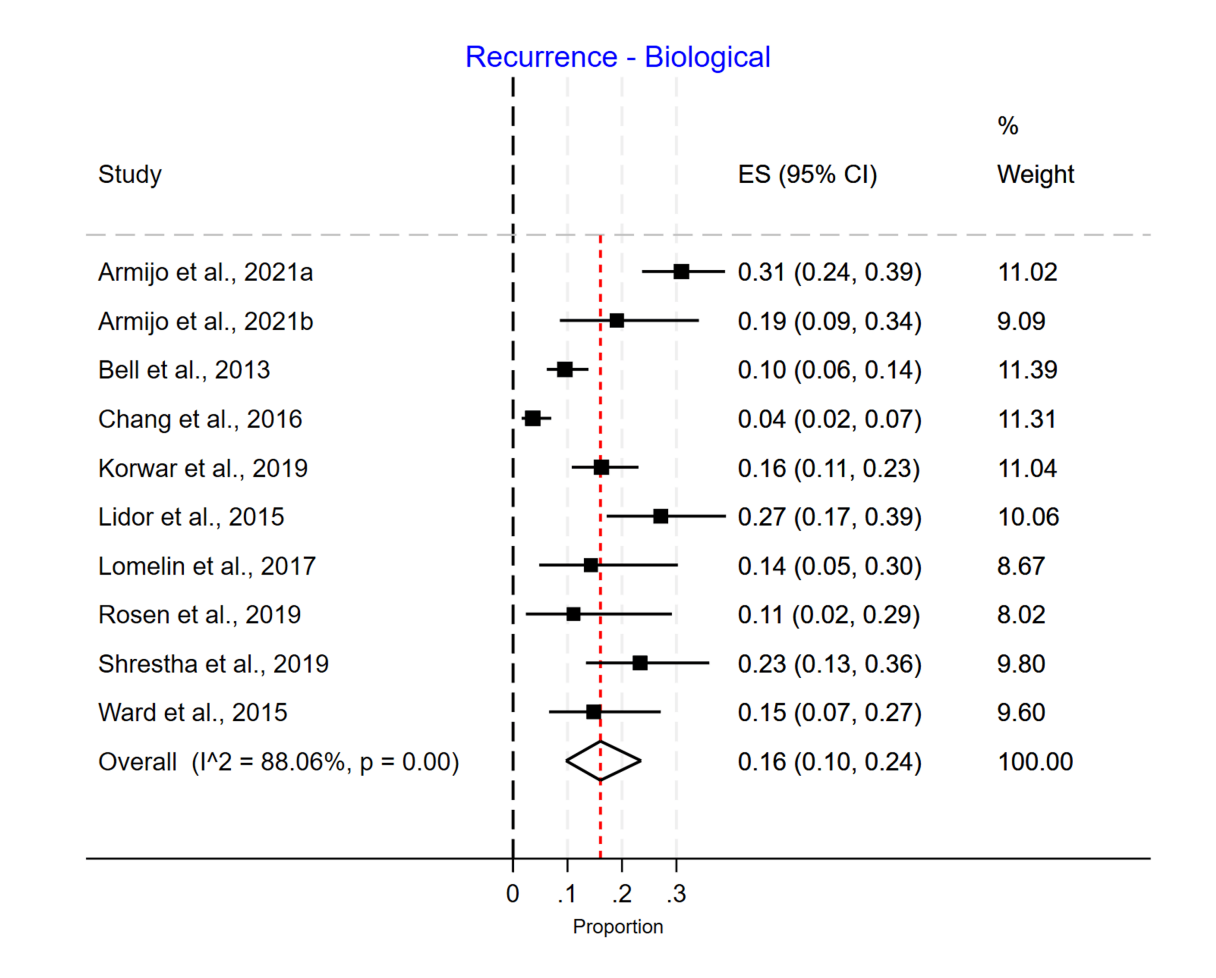
Recurrence rate of biological mesh studies

**Figure 6:**
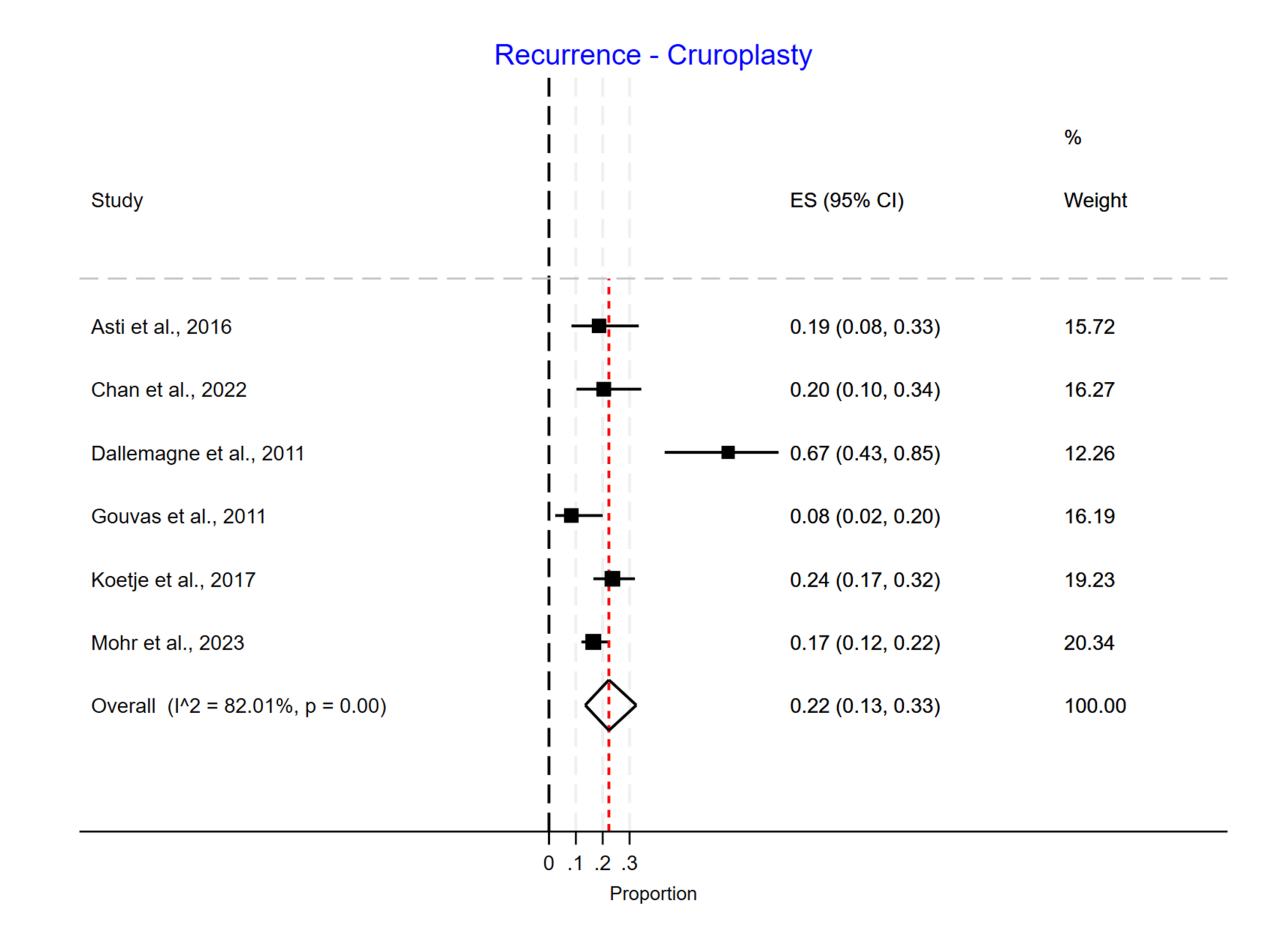
Recurrence rate of suture cruroplasty studies

**Figure 7:**
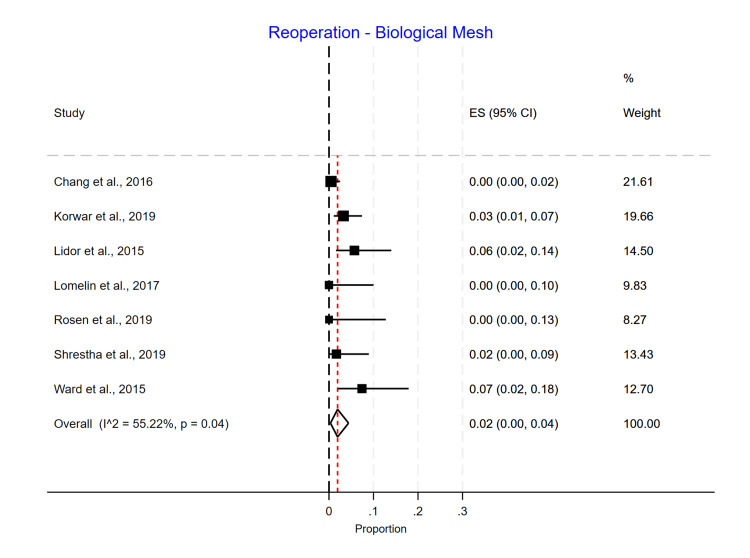
Reoperation rate of the biological mesh group

**Figure 8:**
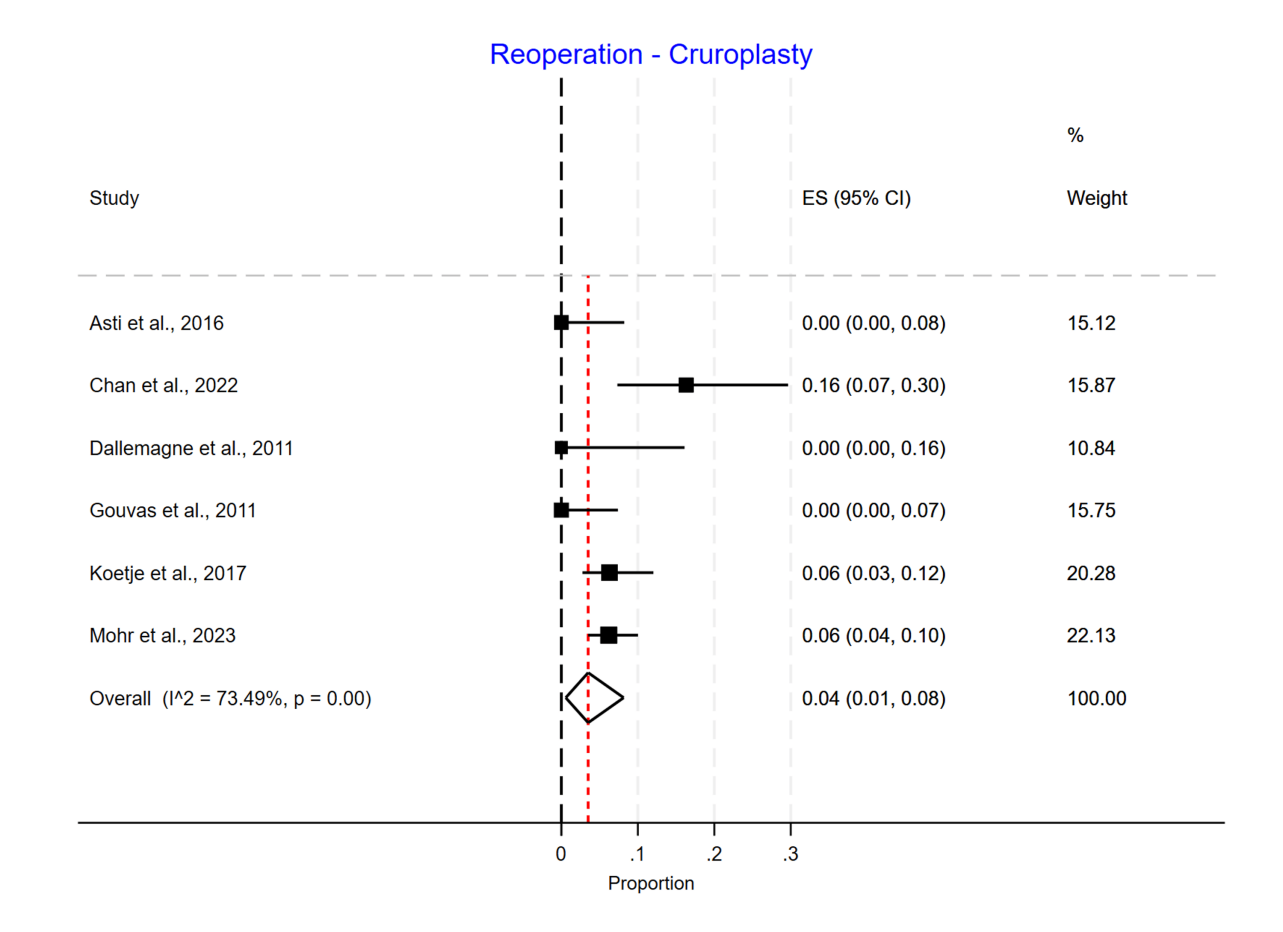
Reoperation rate of the cruroplasty group

Single-arm meta-analyses were conducted for each mesh type, showing that Phasix™ ST mesh [5-7,12] had the lowest recurrence rate of 0.05 (95% CI 0.01-0.10) (Figure 4), followed by biological mesh [2,13-20] 0.16 (95% CI 0.10-0.24) (Figure 5) and suture cruroplasty [21-26] 0.22 (95% CI 0.13-0.33) (Figure 6). No reoperations were reported with Phasix™ ST mesh. The biological mesh had a reoperation rate of 0.02 (95% CI 0.00-0.04) (Figure 7), and cruroplasty had a reoperation rate of 0.04 (95% CI 0.01-0.08) (Figure 8). The Phasix™ mesh was used in four studies, and the pooled analysis did not show significant heterogeneity (I2=44%; p=0.15). However, considerable heterogeneity was found among biological mesh studies reporting recurrence (I2=88%; p<0.001) and cruroplasty studies reporting recurrence (I2=82%; p<0.001). For studies reporting reoperation, heterogeneity was significant for biological mesh (I2=55%; p=0.04) and cruroplasty pooled analyses (I2=73%; p<0.001).

Biological Mesh Type Subgroup Analysis

**Figure 9:**
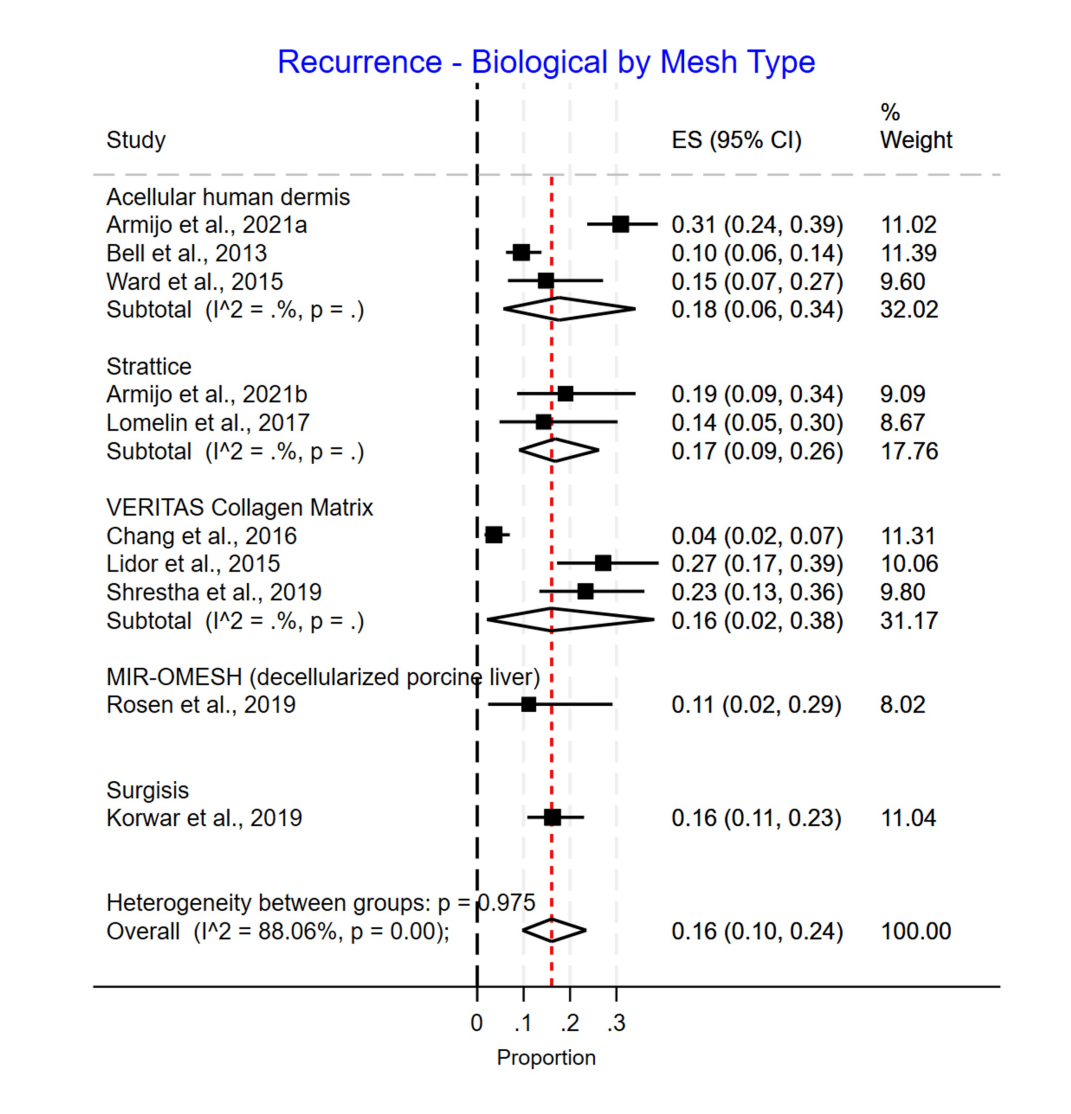
Biological mesh subgroup recurrence rate

For the biological mesh subgroup analysis, we observed that MIROMESH [18] (decellularized porcine liver) had the lowest recurrence rate of 0.11 (95% CI 0.02-0.29), followed by Surgisis [3] and Veritas Collagen Matrix [14,16,19] with a recurrence rate of 0.16 (95% CI 0.11-0.23) and 0.16 (95% CI 0.02-0.38), respectively. This is followed by Strattice mesh [2,17] (0.17; 95% CI 0.09-0.26) and acellular human dermis [2,11,13,20] (0.18; 95% CI 0.06-0.34) (Figure 9).

The reoperation rate for the biological subgroup was as follows: Strattice and MIROMESH (decellularized porcine liver) had similar rates of reoperation 0.00 (95% CI 0.00-0.10) and 0.00 (95% CI 0.00-0.13), respectively, followed by Veritas Collagen Matrix with 0.02 (95% CI 0.00-0.06), Surgisis with 0.03 (95% CI 0.01-0.07), and acellular human dermis with 0.07 (95% CI 0.02-0.18) (Figure 7).

Length of Follow-Up Subgroup Analysis

**Figure 10:**
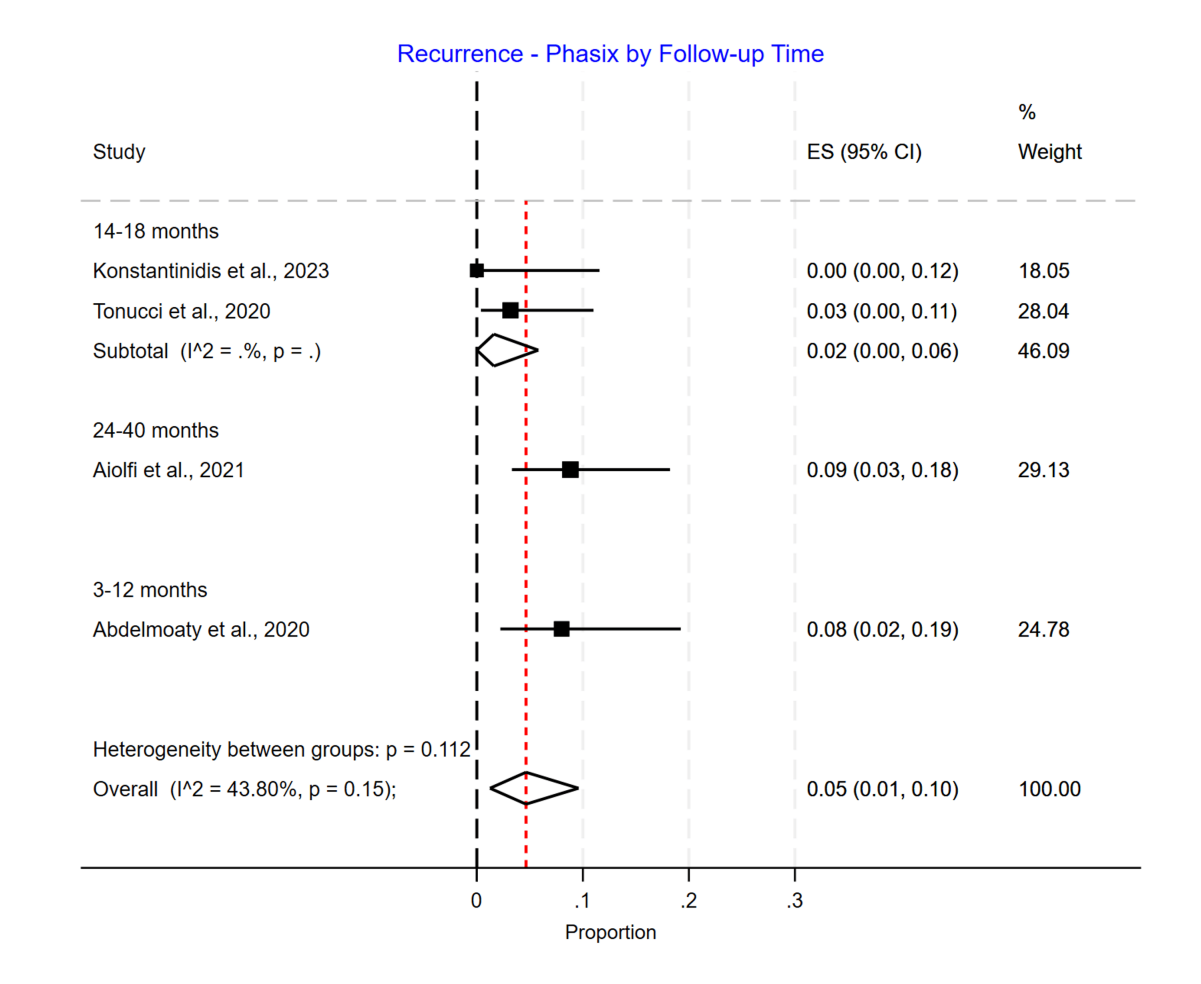
Recurrence rate for Phasix™ mesh based on length of follow-up

As regards subgroup analysis based on the duration of the follow-up for the outcome of recurrence, Phasix™ ST mesh showed no statistically significantly different rates with 0.08 (95% CI 0.02-0.19) for the 3-12-month follow-up, 0.02 (95% CI 0.00-0.06) for the 14-18-month follow-up, and 0.09 (95% CI 0.03-0.18) for the 24-40-month follow-up (p=0.15) (Figure 10).

**Figure 11:**
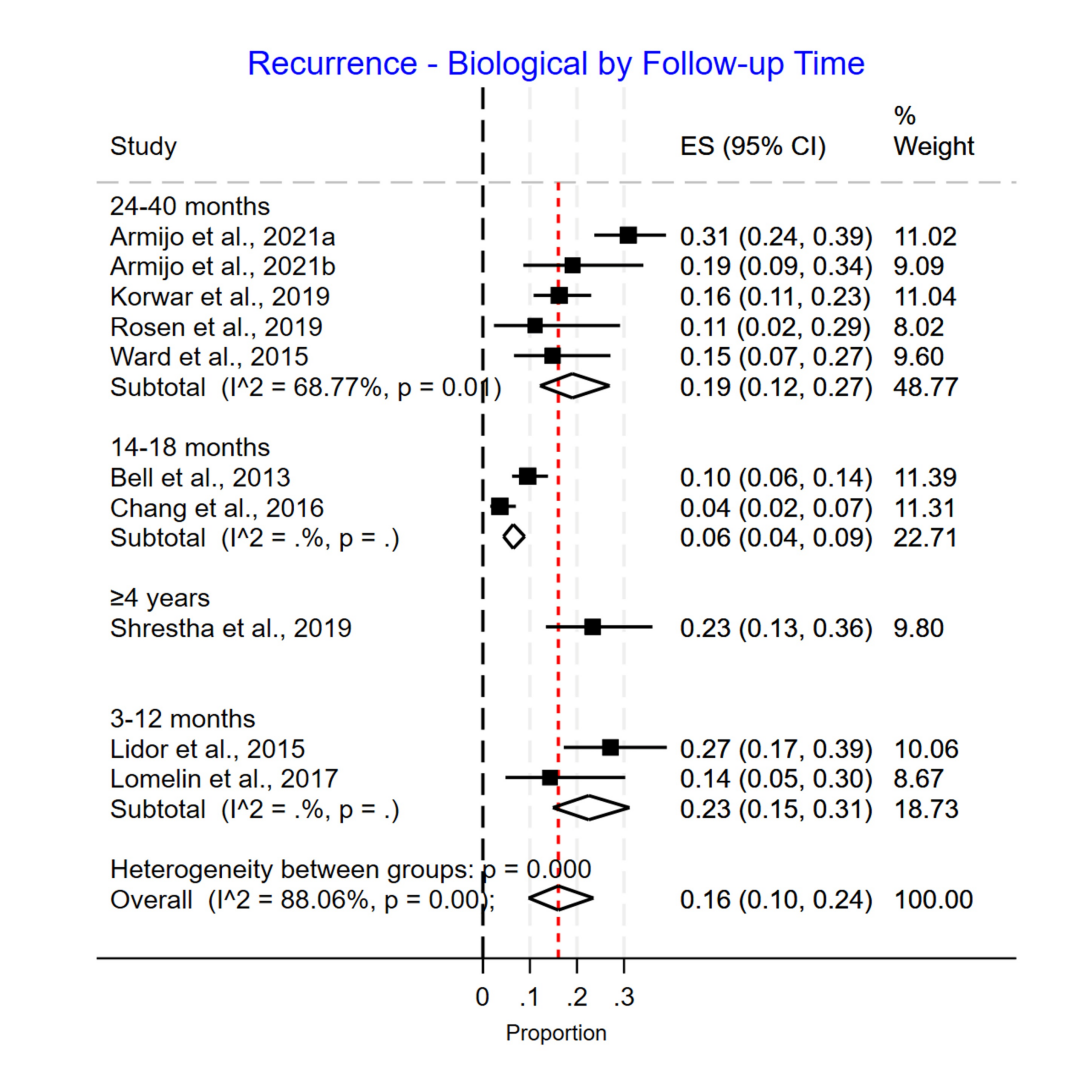
Recurrence rate for biological mesh based on length of follow-up

**Figure 12:**
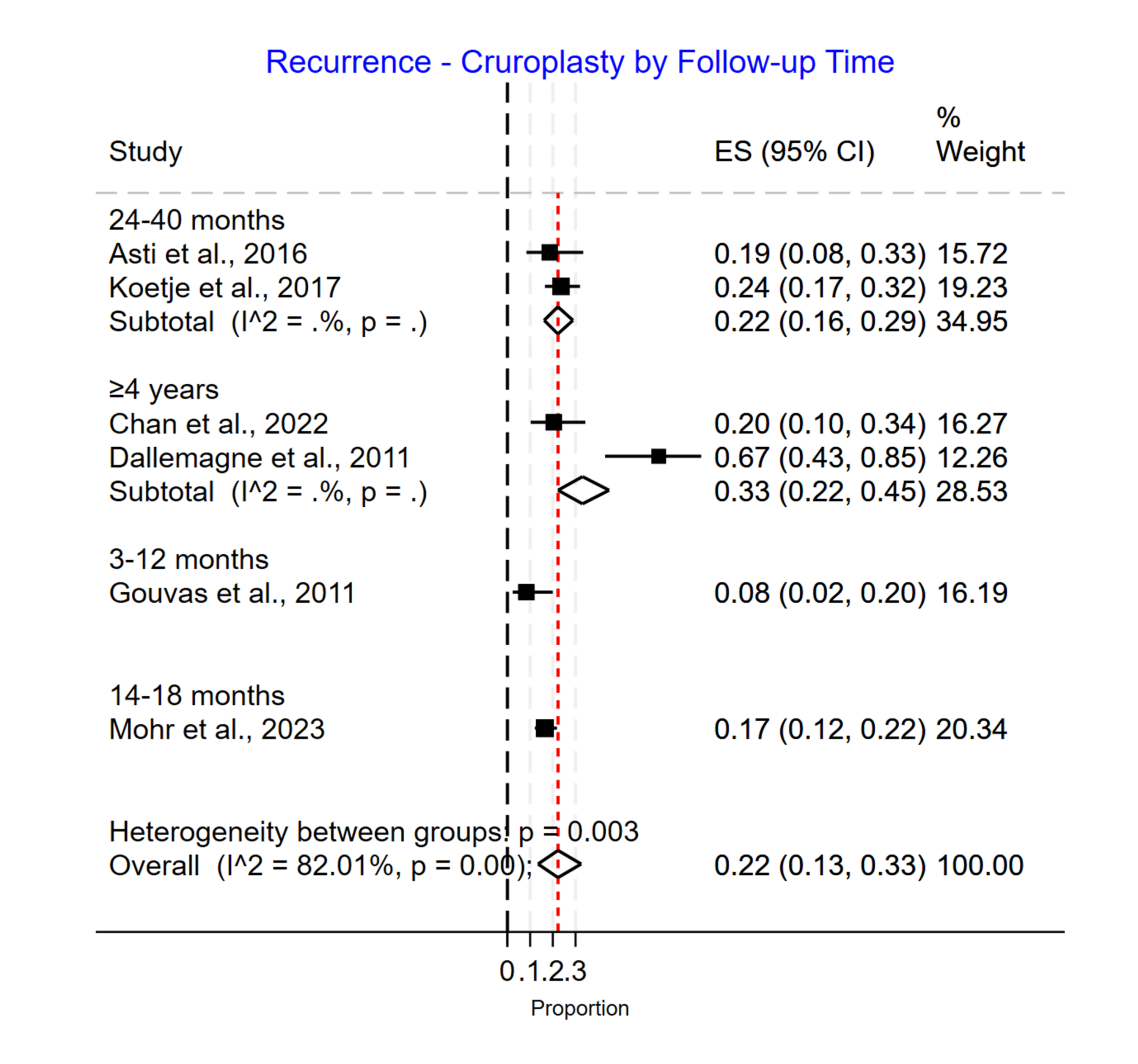
Recurrence rate for cruroplasty based on length of follow-up

For the biological mesh group, the recurrence rates for patients were noticed to be significantly different based on the duration of follow-up with 0.23 (0.15-0.31) for 3-12 months, 0.06 (0.04-0.09) for 14-18 months, 0.19 (0.12-0.27) for 24-40 months, and 0.23 (0.13-0.36) for more than four years of follow-up (p>0.001) (Figure 11). For the suture cruroplasty group, the recurrence rates increased with longer follow-up, with 0.08 (0.02-0.20) for 3-12 months, 0.17 (0.12-0.22) for 14-18 months, 0.22 (0.16-0.29) for 24-40 months, and 0.33 (0.22-0.45) for more than four years of follow-up (p=0.00) (Figure 12).

BMI Subgroup Analysis

**Figure 13:**
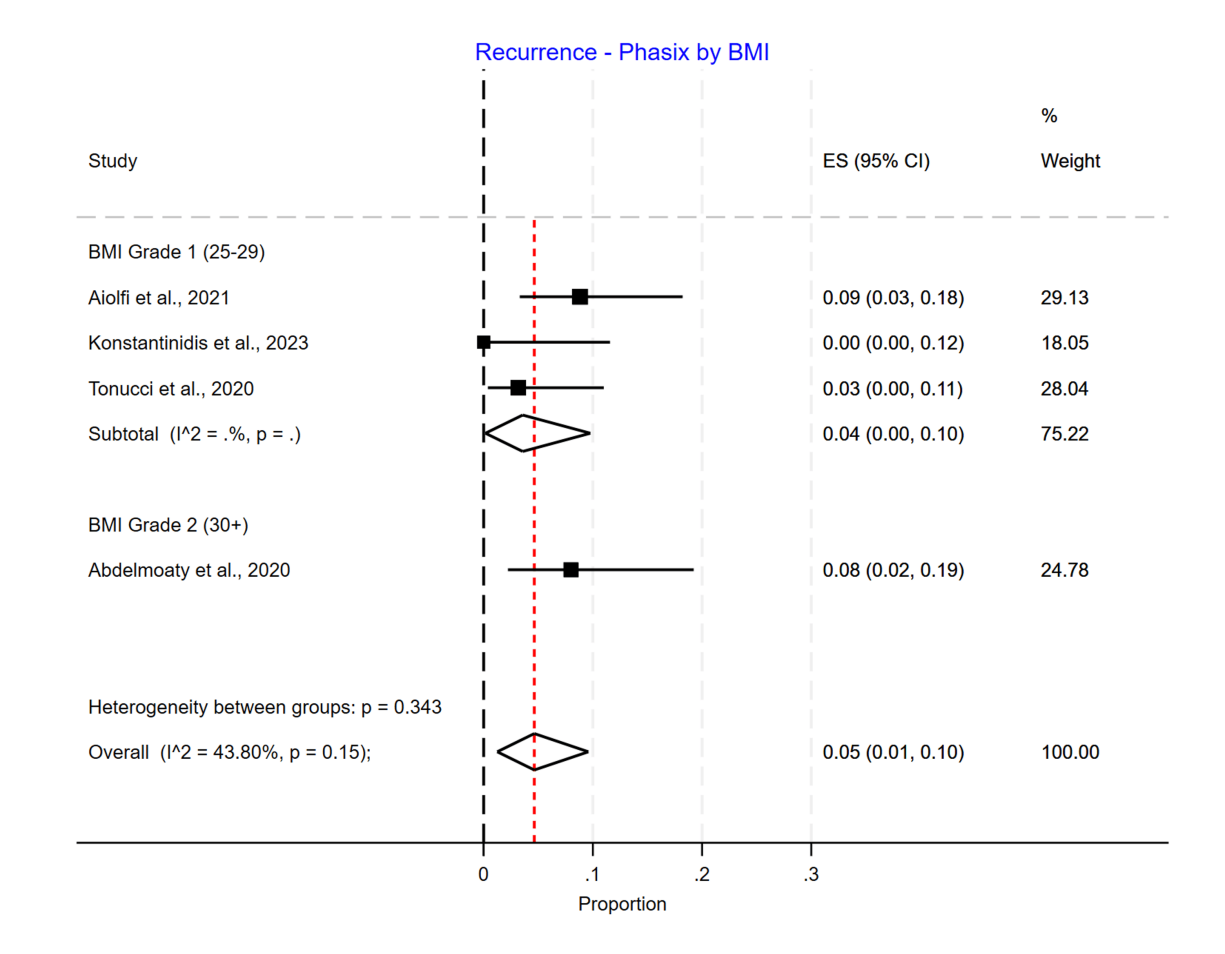
Recurrence rate for Phasix™ mesh based on BMI

**Figure 14:**
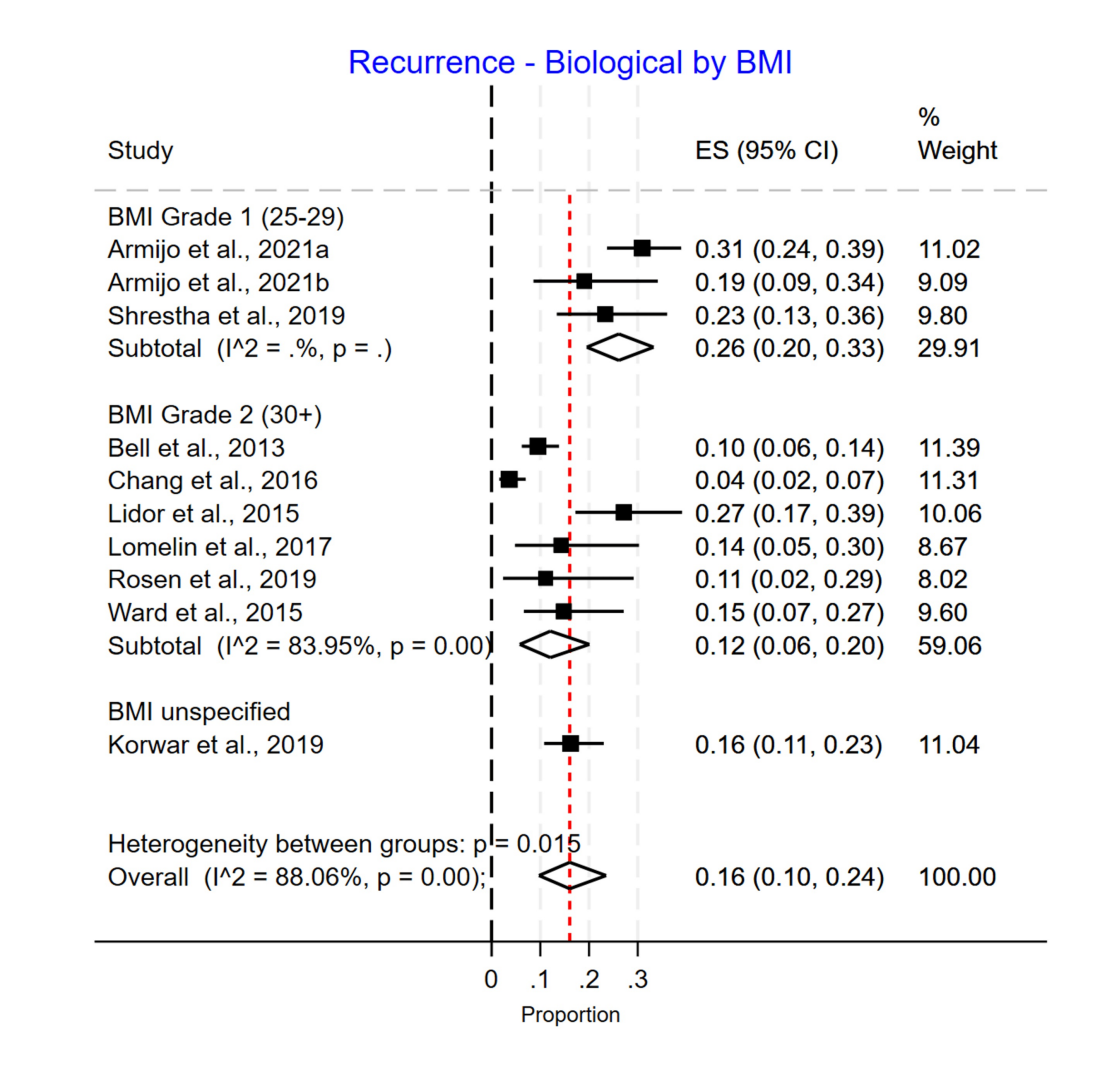
Recurrence rate for biological mesh based on BMI

**Figure 15:**
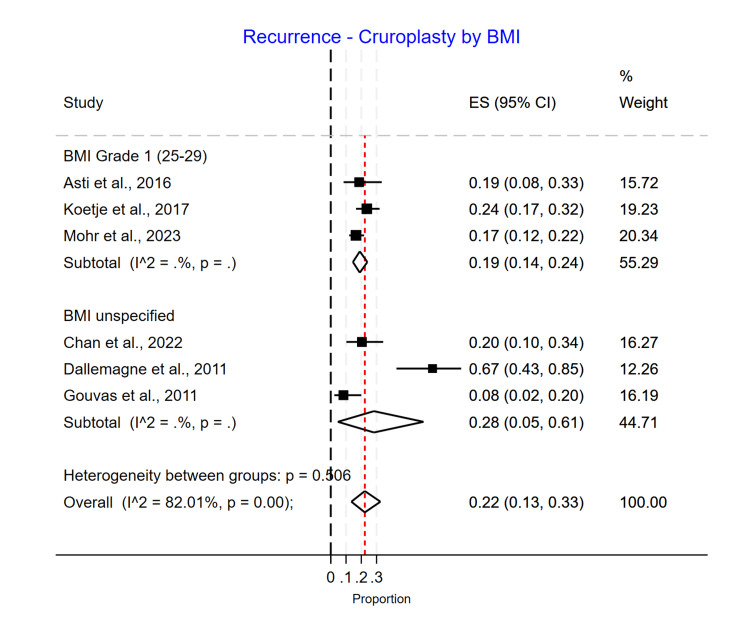
Recurrence rate for cruroplasty based on BMI

In a subgroup analysis by average BMI, the recurrence rate did not significantly differ (p=0.15). However, recurrence appeared to be higher in the Grade 2 BMI group in the Phasix™ ST mesh group, with 0.08 (0.02-0.19) recurrence compared to 0.04 (0.00-0.10) in the Grade 1 BMI group. However, only one study had patients with an average BMI greater than 30 (Grade 2) (Figure 13). For the biological mesh group, the recurrence rate was higher in the Grade 1 BMI group, with 0.26 (0.20-0.33) recurrence compared to 0.12 (0.06-0.20) in the Grade 2 BMI group and 0.16 (0.11-0.23) in the unspecified BMI group (Figure 14). For the suture cruroplasty group, 50% of studies did not report BMI, and the recurrence rate was higher for the unspecified BMI group with 0.28 (0.05-0.61) recurrence compared to 0.19 (0.14-0.24) in the Grade 1 BMI group (Figure 15).

Postoperative Dysphagia

**Figure 16:**
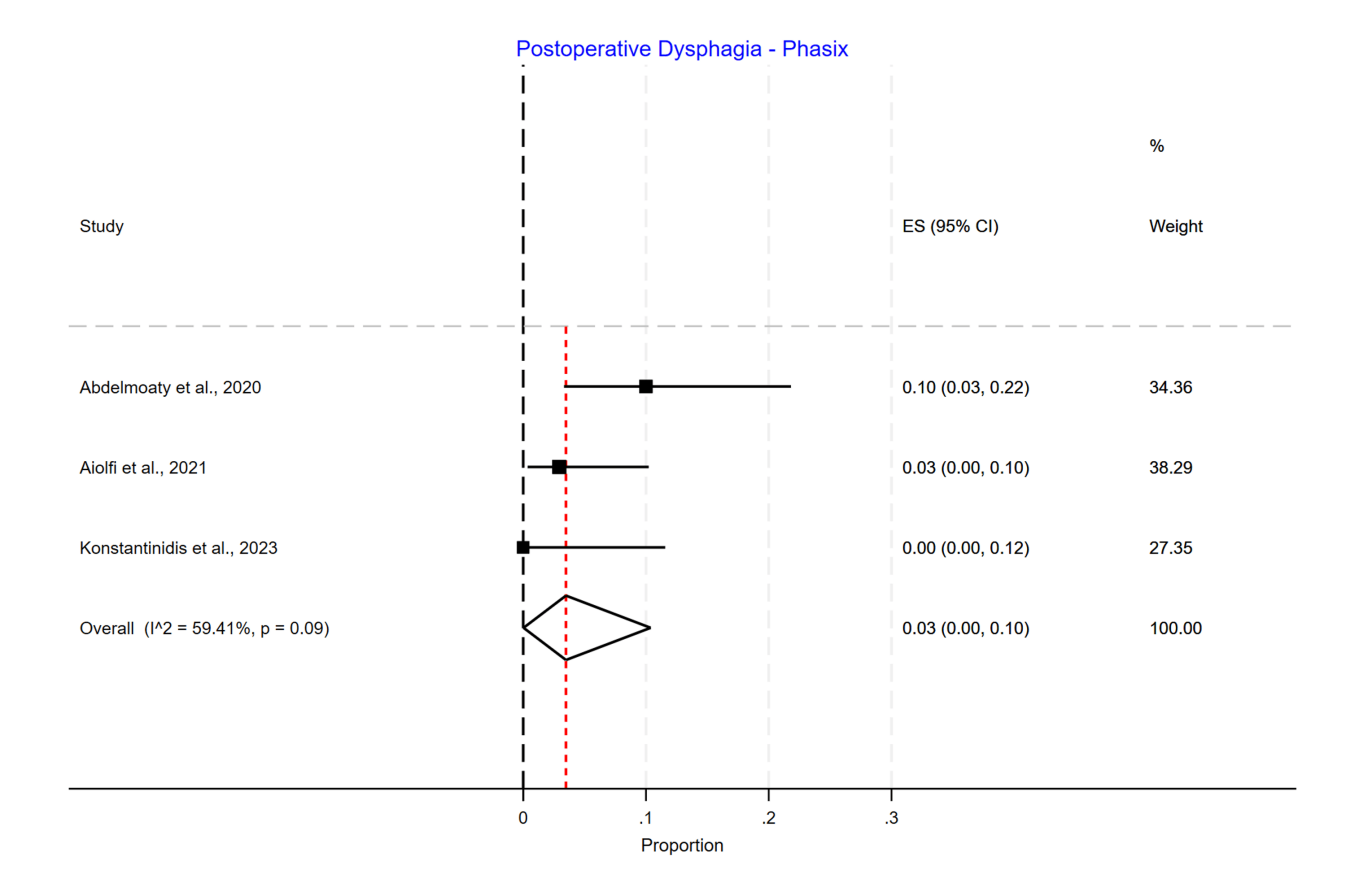
Postoperative dysphagia following Phasix™ mesh repair

**Figure 17:**
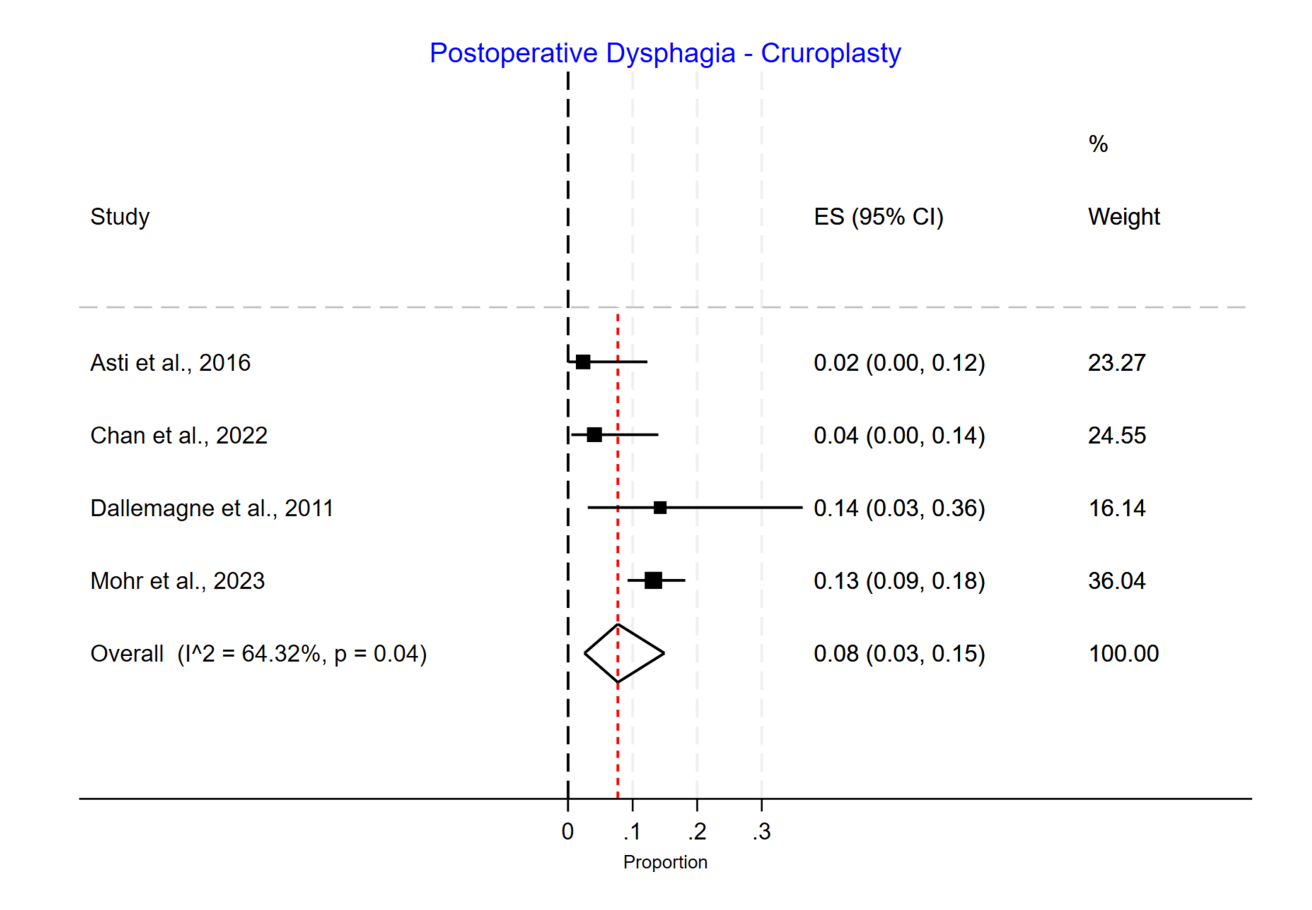
Postoperative dysphagia following cruroplasty repair

**Figure 18:**
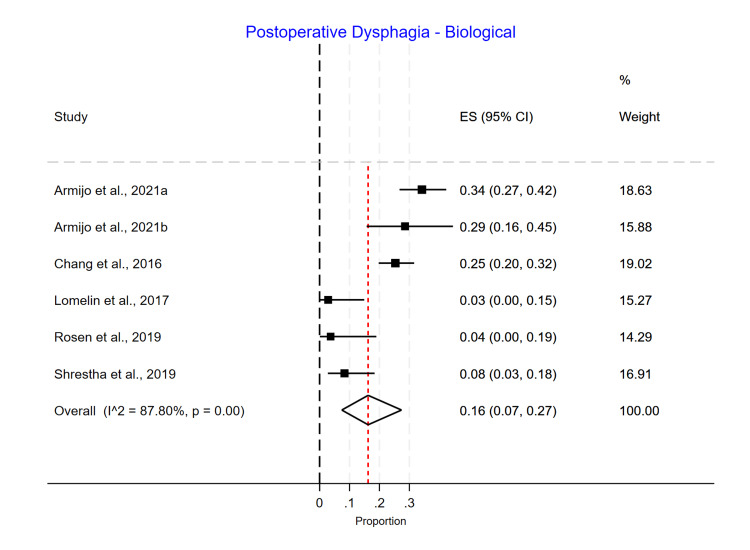
Postoperative dysphagia following biological mesh repair

Phasix™ ST mesh had the lowest dysphagia rate (3%; 95% CI 0.00-0.10) (Figure 16), followed by suture cruroplasty (8%; 95% CI 0.03-0.15) (Figure 17) and biological mesh (16%; 95% CI 0.07-0.27) (Figure 18). Studies reporting postoperative dysphagia showed moderate heterogeneity among Phasix™ mesh (I2=59%; p=0.09) and cruroplasty (I2=64%; p=0.04), but high heterogeneity among biological mesh (I2=88%; p<0.001).

Risk of Bias

To assess the risk of bias, we used the Cochrane risk of bias tool for randomized controlled trials and the ROBINS-I risk of bias tool for non-randomized trials. We believe that the included studies demonstrate fair or better quality (Tables 6-7). Additionally, we believe that the Phasix™ group had a lower follow-up compared to the others, which may have affected the results. We consider this to be a risk of bias.

**Table 6:** Cochrane risk of bias tool for randomized controlled trials

Author	Selection bias: random sequence generation	Selection bias: allocation concealment	Reporting bias: selective reporting	Other sources of bias	Performance bias: blinding (participants and personnel)	Detection bias: blinding (outcome assessment)	Attrition bias: incomplete outcome data
Watson et al. [3]	Unclear: method of random sequence generation not stated	Low risk	Low risk	Low risk	Low risk	Low risk	Low risk

**Table 7:** ROBINS-I risk of bias tool for non-randomized trials GIQLI: Gastrointestinal Quality of Life Index; EGD: esophagogastroduodenoscopy

Author	Bias due to confounding	Bias in the selection of participants	Bias in the classification of interventions	Bias due to deviations from intended interventions	Bias due to missing data	Bias in the measurement of outcomes	Bias in the selection of the reported result	Overall
Abdelmoaty et al. [12]	Low risk	Low risk	Low risk	Unclear: not stated if any procedures were converted to open	Low risk	Low risk	Low risk	Fair
Aiolfi et al. [5]	Low risk	Low risk	Low risk	Unclear: not stated if any procedures were converted to open	Low risk	Low risk	Low risk	Fair
Konstantinidis and Charisis [7]	High risk: comorbidities that increase the intra-abdominal pressure are part of the inclusion criteria but are not elucidated in the paper	Low risk	Low risk	Low risk	High risk: different hernia sizes were recorded, but recurrence rates were compared as one group	Low risk	Low risk	Poor
Panici Tonucci et al. [6]	Low risk	Low risk	Low risk	Low risk	Low risk	Unclear: upper gastrointestinal endoscopy and/or barium/Gastrografin swallow study used for the determination of recurrent hiatal hernia	Low risk	Fair
Armijo et al. [2]	Low risk	Low risk	Low risk	Unclear: not stated if any procedures were converted to open	High risk: retention of only 59.2% of patients at the long-term follow-up	Low risk	Low risk	Poor
Bell et al. [13]	Low risk	Low risk	Low risk	Unclear: not stated if any procedures were converted to open	Low risk	Unclear: recurrence was only evaluated anatomically if patients presented with symptoms, "objective testing" to determine anatomy was stated, but specific methods were not described	Low risk	Fair
Chang and Thackeray [14]	Low risk	Low risk	Low risk	Unclear: not stated if any procedures were converted to open	Low risk	Low risk	Low risk	Fair
Korwar et al. [15]	Low risk	Low risk	Low risk	Low risk	Low risk	Low risk	Low risk	Good
Lidor et al. [16]	Low risk	Low risk	Low risk	Unclear: not stated if any procedures were converted to open	Low risk	Low risk	Low risk	Fair
Lomelin et al. [17]	Low risk	Low risk	Low risk	Low risk	Low risk	Unclear: patients had barium swallow and/or EGD at 12 months postoperatively to assess for recurrence	Low risk	Fair
Oelschlager et al. [10]	Low risk	Low risk	Low risk	Unclear: not stated if any procedures were converted to open	Low risk	Low risk	Low risk	Fair
Rosen et al. [18]	Low risk	Low risk	Low risk	Low risk	Low risk	Low risk	Low risk	Good
Schmidt et al. [11]	High risk: statistically significant age difference between groups	Low risk	Low risk	Low risk	Low risk	Low risk	Low risk	Fair
Shrestha et al. [19]	Low risk	Low risk	Low risk	Low risk	Low risk	Low risk	Low risk	Good
Ward et al. [20]	Low risk	Low risk	Low risk	Low risk	Low risk	Low risk	Low risk	Good
Asti et al. [21]	Low risk	Low risk	Low risk	Low risk	Low risk	Low risk	Low risk	Good
Dallemagne et al. [22]	Low risk	Low risk	Low risk	Low risk	High risk: GIQLI did not exist when the study began, and no preoperative values were collected as a result	Low risk	Low risk	Fair
Chan et al. [23]	Low risk	Low risk	Low risk	Unclear: not stated if any procedures were converted to open	Low risk	Low risk	Low risk	Fair
Gouvas et al. [24]	Low risk	Low risk	Low risk	Low risk	High risk: different hernia sizes were recorded, but recurrence rates were compared as one group	Low risk	Low risk	Fair
Koetje et al. [25]	High risk: a statistically significant difference in the American Society of Anesthesiologists Physical Status scores between groups	Low risk	Low risk	Low risk	Low risk	Low risk	Low risk	Fair
Mohr et al. [26]	High risk: statistically significant age difference between groups	Low risk	Low risk	Low risk	Low risk	Unclear: the method of assessing radiographic recurrence is not stated, and patients were not evaluated for radiographic recurrence if asymptomatic	Low risk	Poor

Discussion

We conducted a meta-analysis review study comparing Phasix™ ST bioabsorbable mesh to biological mesh. We compared both of these options to primary repair (suture cruroplasty) in terms of recurrence rate, reoperation rate, and postoperative dysphagia. We analyzed 22 articles involving 2,008 patients who received different types of mesh or suture cruroplasty alone.

Our study found that the use of bioabsorbable mesh (Phasix™ ST mesh) for hiatal hernia repair resulted in a lower recurrence rate (5%) compared to biological mesh (16%) and suture cruroplasty (22%). We found no reoperation cases with the Phasix™ ST mesh, while the reoperation rates were 2% for biological mesh and 4% for suture cruroplasty. The recurrence rate was the lowest for Phasix™ ST mesh at all points of follow-up. In the biological mesh group, we observed a higher incidence of recurrence in the first year and after more than four years compared to intermediate follow-up times. Additionally, in the suture cruroplasty group, the incidence of recurrence increased over time. The biological mesh subgroup showed a higher recurrence incidence than Phasix™ ST mesh and a lower incidence compared to suture cruroplasty. Regarding the relationship between BMI and recurrence rate, we believe that we cannot rely on our results to determine the effect of BMI on the recurrence rate because many studies do not mention the mean BMI (unspecified BMI).

Recurrence Rate of Hiatal Hernia

A meta-analysis revealed that the recurrence rate of hiatal hernia was found to be higher in patients undergoing suture cruroplasty alone (42%) compared to repair with mesh (9%), but it was not stated whether these findings were statistically significant [27]. This literature supports our meta-analysis findings, which demonstrated a lower recurrence rate in hernia mesh repair, whether using Phasix™ ST or biological mesh, compared to cruroplasty.

Bioabsorbable Phasix™ ST Mesh Recurrence Versus Biological Mesh Recurrence

Limited studies on the use of Phasix™ ST mesh in hiatal hernia repair have shown recurrence rates between 0% and 9%, without significant differences [5-7,12]. The studies with biological mesh reported recurrence rates between 4% and 31%. The included biological mesh studies demonstrated significant differences in the overall recurrence rate, likely due to the various types of biological mesh used in each study. Our meta-analysis found that even within the subgroup of biological mesh, there was a significant difference in recurrence rates between mesh types (p=0.00). This highlights the importance of prospective studies comparing each specific type of biological mesh with Phasix™ ST mesh. Regardless, our included Phasix™ ST mesh studies showed lower recurrence rates than those observed with biological mesh. 

Phasix™ ST mesh repair has demonstrated significantly higher strength compared to the natural abdominal wall at different time points after implantation. For example, in a porcine model, differences between the natural abdominal wall and Phasix™ ST mesh strength of reinforcement were as follows: 80% greater strength at eight weeks, 65% greater strength at 16 weeks, 58% greater strength at 32 weeks, and 37% greater strength at 48 weeks [28]. Moreover, Phasix™ ST mesh plays a crucial role in the initial healing phase, providing essential strength with rapid tissue ingrowth and vascularization due to its open-pore monofilament structure [29].

Reoperation Rate Following Hiatal Hernia Repair

We found that the reoperation rate was lower in the biological mesh group compared to the non-mesh group (suture cruroplasty) [3,10,11]. However, it is important to note that there was only a statistically significant difference between groups in one study [11]. As confirmed in our study, in all four studies where Phasix™ ST mesh was used for hiatal hernia repair, there was a 0% reoperation rate [4-7], compared to the biological mesh group, where the reoperation rate ranged from 2% to 7% [14-20], with an overall reoperation rate of 2%. For suture cruroplasty, we found a range of reoperation rates from 0% to 16%, with an overall reoperation rate of 4%. Additionally, in the double-arm meta-analysis, the randomized and non-randomized trials comparing biological vs. cruroplasty showed higher odds of reoperation among the cruroplasty group.

Postoperative Dysphagia Following Hiatal Hernia Repair

In our study, we found that postoperative dysphagia in the Phasix™ ST mesh group ranged from 0% to 10%, with an overall incidence of 3% without a significant difference between the studies. For studies using biological mesh, the postoperative dysphagia ranged from 3% to 34%, with a substantial difference between the studies (p=0.00) and an overall incidence of 16%. Regarding suture cruroplasty, we found that postoperative dysphagia ranged from 2% to 14%, with an overall incidence of 8% and a significant difference between the studies (p=0.04).

Limitations

Our meta-analysis has a few limitations. During our extensive research on hiatal hernia projects, we observed discrepancies in how hernia size is reported. We believe the existing literature requires new methods for reporting hiatal hernia to establish a consensus and promote a uniform approach to reporting hernia size. Another limitation includes the unavailability of data for BMI in many studies, which makes it difficult to conclude the effect of BMI on recurrence rate and reoperation. Accurate information regarding factors such as age, sex, the type of fundoplication, and other technical issues was lacking and varied between the studies. Moreover, in the Phasix™ mesh group, we found only one study with a follow-up of more than two years (24-40 months). We need longer follow-up periods to establish the possibility of late recurrence. We strongly believe that this issue has a significant impact when comparing hernia recurrences, reoperations, and complications and needs to be accurately addressed in future studies.

## Conclusions

Phasix™ ST mesh showed the lowest recurrence, reoperation, and dysphagia rates compared to biological mesh and suture cruroplasty in hiatal hernia repair, making it a preferred option. However, our double-arm meta-analysis between patients who underwent suture cruroplasty and those who had reinforcement of the hiatal hernia repair with biological mesh showed no significant difference in recurrence and reoperation rates. 
